# Rapidly Progressive Dementia: Predictive Clues and a Proposed Diagnostic Approach Focused on Neurodegenerative Causes

**DOI:** 10.7759/cureus.96097

**Published:** 2025-11-04

**Authors:** Francisco Gortari, Maria T Rosero, Conrado J Simison, Laura Ottone, Raul C Rey, Mayra Aldecoa, Carlos S Claverie, Guido Dorman

**Affiliations:** 1 Neurology, Jose María (JM) Ramos Mejia Hospital, Buenos Aires, ARG; 2 Stroke Center, Favaloro Foundation, Buenos Aires, ARG; 3 Memory Clinic, Institute of Cognitive Neurology (INECO), Buenos Aires, ARG

**Keywords:** aducanumab, lecanemab, neurodegenerative diseases, predictive score, rapidly progressive dementia, tau biomarkers

## Abstract

Introduction

Rapidly progressive dementia (RPD) is a clinical challenge due to the relatively high prevalence of potentially reversible causes, some of which have specific treatments. Consequently, the diagnostic approach is broader than in slowly progressive dementias. Nevertheless, neurodegenerative (ND) diseases remain a leading etiology in most RPD cohorts. Underreporting of prior cognitive decline contributes to extensive testing, higher costs, longer diagnostic timelines, and invasive procedures, often without identifying causes beyond ND pathology.

Objective

This study aims to evaluate the predictive utility of factors such as age of onset, symptom duration, absence of neurological signs, and absence of crisis for identifying ND etiologies in patients with RPD. Therefore, we aim to identify a subgroup of patients for whom the diagnostic and therapeutic approach could be optimized, facilitating an accurate diagnosis and improving the prognosis and treatment of these cases.

Materials and methods

A retrospective, observational, cross-sectional study was conducted of 135 diagnoses, with RPD patients treated at a public hospital in Buenos Aires (2013-2024). Data included demographics, comorbidities, clinical features, diagnostic delay, laboratory and imaging findings, final diagnosis, follow-up, and outcomes.

Quantitative data were summarized using means or medians, while categorical data were expressed as percentages. R and RStudio software (version 4.3.2, October 31, 2023) (Posit Software, Boston, MA, US) were used for variable analysis.

Results

A comprehensive analysis was performed on 135 patients diagnosed with RPD, aiming to develop an algorithm to identify ND diseases as the underlying etiology. Of these, 30 patients were excluded after the initial diagnostic evaluation revealed a definite cause. Among the remaining 105 patients, an additional 35 were excluded because imaging findings suggested a non-ND etiology (such as temporal, thalamic, insular, frontal, and cortical hyperintensities on diffusion-weighted imaging (DWI), T2, and fluid-attenuated inversion recovery (FLAIR) or contrast enhancement).

In the remaining 70 patients, the predictive value of selected variables, such as age, symptom duration, absence of neurological signs, and crisis, was evaluated. Among these, age and symptom duration showed significant predictive value for ND etiology (sensitivity (Se) 55%, specificity (Sp) 75%, positive predictive value (PPV) 72%, negative predictive value (NPV) 58%; p = 0.02). Logistic regression, including age, disease progression, and absence of neurological signs, identified age as an independent predictor (p = 0.004). Model performance was moderate (area under the curve (AUC) 0.74; 95% confidence interval (CI) 0.63-0.86). Finally, when applying the model to the cohort, 11 patients were identified, all with ND etiology.

Conclusion

Considering these factors, ND disorders-historically viewed as diagnoses of exclusion-may become a central component of the diagnostic approach to RPD, alongside autoimmune encephalitis (AE). We propose the development of an algorithm based on a predictive model that allows for the identification of a subgroup of patients in whom the diagnostic approach can be optimized, avoiding unnecessary studies and prioritizing the early identification of a possible ND etiology.

## Introduction

Dementia is defined in DSM-IV (Diagnostic and Statistical Manual of Mental Disorders, 4th ed.) as a decline in one or more cognitive domains sufficient to interfere with independence in everyday activities [1]. The major types of dementia include Alzheimer’s disease (AD), frontotemporal dementia (FTD), dementia with Lewy bodies (LBD), vascular dementia, and less common etiologies such as prion diseases or metabolic disorders [2]. Causes can be broadly divided into neurodegenerative (ND) (e.g., AD, FTD, LBD, and prion diseases) and non-ND (e.g., vascular, autoimmune, infectious, toxic-metabolic, and neoplastic) [2].

Rapidly progressive dementia (RPD) develops within one to two years from the onset of the first symptom until dementia onset, with progression frequently occurring in weeks or months [3,4]. Initially associated with prion disease, such as Creutzfeldt-Jakob disease (CJD), the spectrum of RPD is now focused on autoimmune encephalitis (AE), a clinical challenge since early diagnosis allows timely treatment, which may alter the prognosis [3,4].

The scientific literature reports numerous studies on the diagnostic approach in patients with RPD, suggesting the need for exhaustive evaluations using laboratory and imaging studies as an initial screening for treatable causes [3,4]. In addition, lumbar puncture is required to complete the diagnostic assessment, which entails hospitalization, invasive procedures, and high healthcare costs [3,4].

On the other hand, the most common ND diseases, such as AD, LBD, and FTD, usually manifest with an insidious onset and slow progression, with a disease duration exceeding five years [5]. However, these causes can sometimes present as RPD, especially in older patients or those with a prolonged course of disease. In addition, the absence of seizures and focal neurological signs (aside from extrapyramidal and cognitive symptoms) has been associated with a higher probability of ND etiology in these cases [5,6].

In cohorts of patients with RPD, ND diseases constitute a major etiological group, with prevalence rates consistently exceeding 30%. Van Everbroeck et al. analyzed a cohort of 201 patients (70 men and 62 women; age range, 31-91 years) and identified ND etiologies in 39% of cases, underscoring the diagnostic complexity of RPD, which often involves extensive investigations and may still yield no definitive cause beyond ND pathology [7]. Similarly, in the Athens cohort (n = 68; age range, 35-83 years), ND etiologies accounted for 47.1% of cases, while in the Barcelona cohort (n = 49; age range, 41-86 years), the prevalence was 36.8% [3]. More recently, Cubas Guillen et al. conducted a meta-analysis including 1,006 patients and confirmed ND disorders as the most frequent underlying cause [8].

Traditionally, this group has received less relevance in the context of RPD since the diagnosis is established by exclusion [3]. However, recent advances in precise serum biomarkers [9,10] and the development of targeted therapies, such as lecanemab and aducanumab, recently approved by the FDA [11,12], have opened new diagnostic and therapeutic possibilities for these patients. This suggests a future in which the approach to RPD from an ND perspective could acquire equal relevance to autoimmune etiologies.

This study aims to evaluate the predictive utility of factors such as age of onset, disease progression, and the presence of non-cognitive symptoms for the identification of ND etiologies in patients with RPD. Therefore, we aim to identify a subgroup of patients for whom the diagnostic and therapeutic approach could be optimized, facilitating an accurate diagnosis and improving the prognosis of these cases.

## Materials and methods

Study design

**Figure 1:**
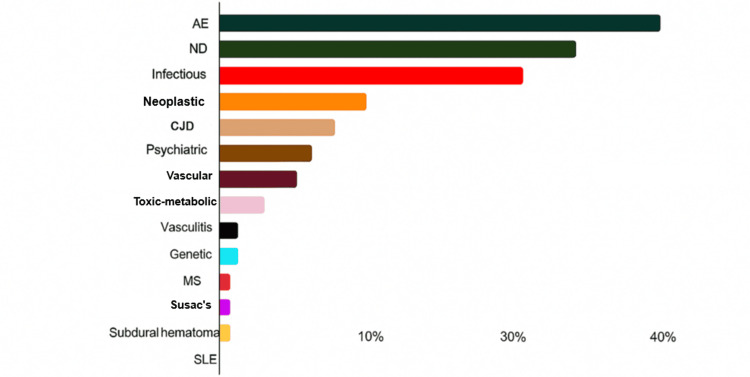
Percentage distribution of different etiologies in patients with RPD (N = 135) AE: autoimmune encephalitis; ND: neurodegenerative; CJD: Creutzfeldt-Jakob disease; MS: multiple sclerosis; SLE: systemic lupus erythematosus; RPD: rapidly progressive dementia Image credits: Francisco Gortari

A retrospective, observational, cross-sectional study was conducted using data from medical records of a public hospital in the city of Buenos Aires. The study covered the period from 2013 to 2024 and included a comprehensive analysis of 135 patients diagnosed with RPD (Figure 1). The study was approved by the institutional ethics committee (approval number No. 01/2025), and informed consent was obtained from all participants or their legal representatives.

Inclusion criteria

Adult patients diagnosed with RPD between 2013 and 2024 were included. RPD was defined as the progression from the first cognitive symptom to established dementia within ≤2 years, regardless of etiology [3]. Dementia was diagnosed according to the Diagnostic and Statistical Manual of Mental Disorders, Fourth Edition, Text Revision (DSM-IV-TR), requiring (i) evidence of significant cognitive decline from a previous level of performance in at least one cognitive domain, (ii) interference with independence in daily activities, (iii) absence of delirium, and (iv) exclusion of other mental disorders [1].

Exclusion criteria

The exclusion criteria include the following: patients with cognitive decline evolving over a period longer than two years, not fulfilling the definition of RPD; patients with cognitive impairment attributable to acute or reversible conditions (e.g., delirium); and patients with incomplete clinical, brain magnetic resonance imaging (MRI), laboratory, or follow-up data that precluded diagnostic classification.

Data collection

Data were extracted from medical records and included the following variables: demographic characteristics, comorbidities, clinical presentation, time from symptom onset to diagnosis, final diagnosis and follow-up, and outcomes. All patients underwent brain MRI (1.5 or 3 T), which was reviewed by a specialist. All patients were studied with routine blood tests and a serologic test for human immunodeficiency virus and syphilis. Additional serologic tests were performed as clinically indicated, including vitamin B12 and folate levels, as well as hepatitis B and C serologies. When clinically indicated, a lumbar puncture was performed if no contraindications were found. In patients with a suspected diagnosis of AE, antibodies were tested. Neuropsychological screening was also conducted using either the Montreal Cognitive Assessment (MoCA, cut-off <18) or the Mini-Mental State Examination (MMSE, cut-off < 24).

Statistical analysis

Statistical analyses were performed using R and RStudio software (version 4.3.2, released on October 31, 2023) (Posit Software, Boston, MA, US) [13]. The variables age (older age), symptom duration (longer duration), absence of neurological signs, and absence of seizures were selected based on their clinical or demographic nature and their previously reported association with ND etiology, as noted above [5,6].

The distribution of quantitative variables was analyzed using graphical and analytical methods (Shapiro-Wilk test). Parametric tests, such as the Student t-test for normal distribution and the Mann-Whitney test for non-normal distribution, were used. For qualitative variables, the chi-squared test or Fisher's test was used when necessary. Subsequently, a logistic regression analysis was performed, including all three variables. A p-value < 0.05 was considered significant. Quantitative data were summarized using means or medians, depending on distribution, while categorical data were presented as percentages.

## Results

**Figure 2:**
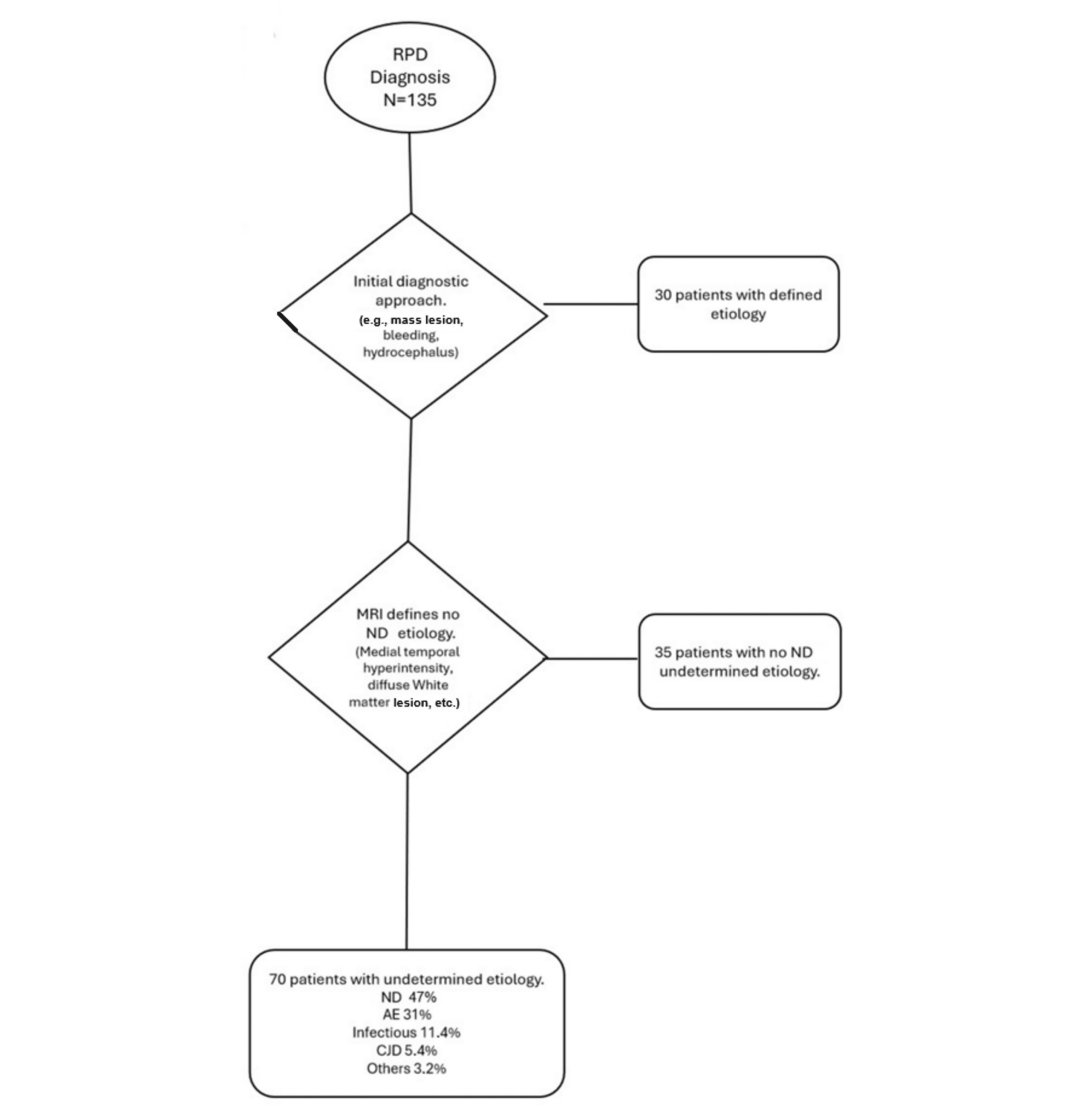
Exclusion criteria of non-ND etiology (N = 135) RPD: rapidly progressive dementia; AE: autoimmune encephalitis; ND: neurodegenerative; CJD: Creutzfeldt-Jakob disease; MRI: magnetic resonance imaging Image credits: Guido Dorman

A total of 135 patients with a final diagnosis of RPD were included: 73 men and 62 women, with a median age of 64 years (range 17-91) and a median symptom duration of 6.2 months. The algorithm excluded 30 patients who received a definitive diagnosis after the initial diagnostic approach (including complete laboratory tests, neuropsychological screening evaluation, and MRI), reducing the sample size to 105. From this group, 35 patients whose neuroimaging, although not providing a specific diagnosis, ruled out an ND etiology (such as cortical, basal ganglia, and thalamic hyperintensities on diffusion-weighted imaging (DWI); temporal, insular, frontal, and cortical T2 and fluid-attenuated inversion recovery (FLAIR) hyperintensities; and contrast enhancement) were also excluded. The final sample consisted of 70 patients, with the following etiological distributions: ND in 47% (n = 33), AE in 31% (n = 22), infectious causes in 11.4% (n = 8), CJD in 5.4% (n = 4), and other etiologies in 3.2% (n = 3) (Figure 2).

Predictive demographic and clinical factors-including age at onset, disease progression, absence of neurological signs such as ataxia, myoclonus, paresis, hypoesthesia, aphasia, visual field defect, dystonia, and stereotypies (excluding cognitive or extrapyramidal symptoms typical of ND diseases), and the presence of seizures reported in previous studies-were evaluated to differentiate ND diseases from non-ND in patients with RPD [5,6]. Univariate analysis of continuous variables revealed statistically significant age differences (p = 0.001) and time of onset (p = 0.024) (Table 1). Patients with ND diseases had a median age of 72 years, while those without had a median age of 63 years. Symptom duration to consultation averaged 12 months for patients with ND diseases and three months for the others.

**Table 1:** Predictors of ND etiology Univariate analysis of quantitative variables using the Mann-Whitney method

	Non-neurodegenerative	Neurodegenerative (ND)	Test statistic	p-value	Method
N (70)	37	33	-	-	-
Age	63.00 (52.00, 71.00)	72.00 (64.00, 81.00)	-	0.001	Mann-Whitney
Symptom duration (months)	3.00 (1.00, 12.00)	12.00 (5.00, 12.00)	-	0.024	Mann-Whitney

Based on the previous results, we analyzed the variables as categorical (age > 65 years, symptom duration >6 months, presence of neurological signs, and seizures). Statistically significant associations were found for age and symptom duration, whereas no significance was observed for the absence of neurological signs or for seizures (Table 2).

**Table 2:** Age, symptom duration, crisis, and absence of neurological signs as categorical variables ND: neurodegenerative; NPV: negative predictive value; PPV: positive predictive value

	% ND	Test statistic	p-value	Method	Sensitivity	Specificity	NPV	PPV
Over 65	63.2	-	0.02	Fisher’s test	55%	75%	58%	72%
Symptom duration greater than 6 months of evolution	65.8	-	0.02	Fisher’s test	55%	75%	58%	72%
Absence of neurological signs	75.9	0.483	0.487	Chi square	75%	24.30%	52.9%	47.2%
Crisis	0	2.24	0.137	Chi square	0%	85%	49.2%	0%

**Figure 3:**
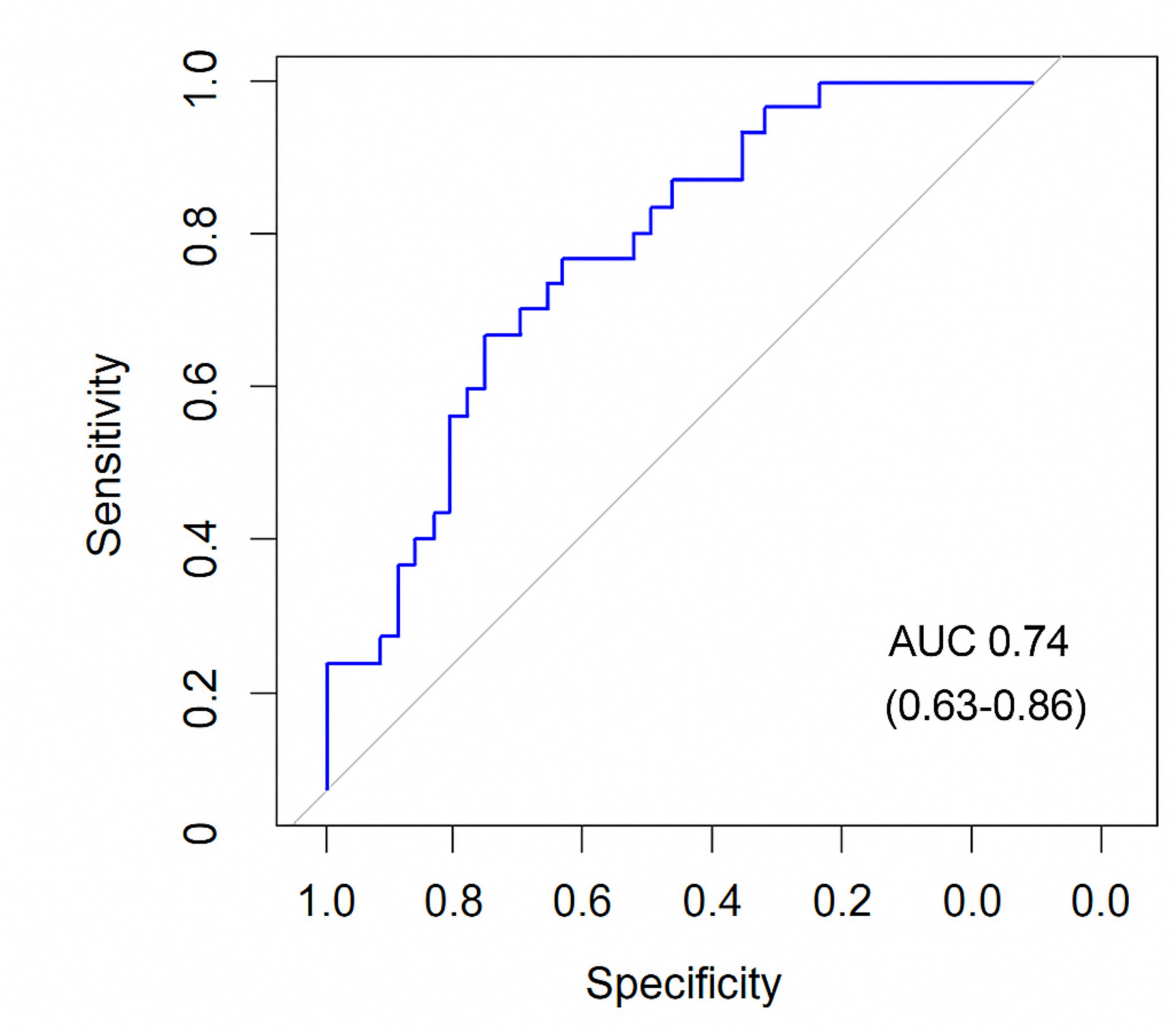
ROC curve generated from the model including age, symptom duration, and absence of neurological signs AUC: area under the curve; ROC: receiver operating characteristic Image credits: Carlos S. Claverie

A logistic regression analysis was performed, including age, disease progression, and absence of neurological signs as variables. The results showed that age was an independent predictor of ND etiology (p = 0.004) (Table 3). To evaluate the model performance, a receiver operating characteristic (ROC) curve was generated, resulting in an area under the curve (AUC) of 0.74 (0.63-0.86), indicating moderate performance (Figure 3).

**Table 3:** Logistic regression model including three variables CI: confidence interval

Predictors	Odds ratios	CI	Std. error	p-value	Method
Age	1.07	(1.03-1.13)	0.03	0.004	Logistic regression
Symptom duration greater than 6 months of evolution	1.04	(0.97-1.12)	0.04	0.279	Logistic regression
Absence of neurological signs	2.02	(0.5-8.71)	1.44	0.327	Logistic regression

**Figure 4:**
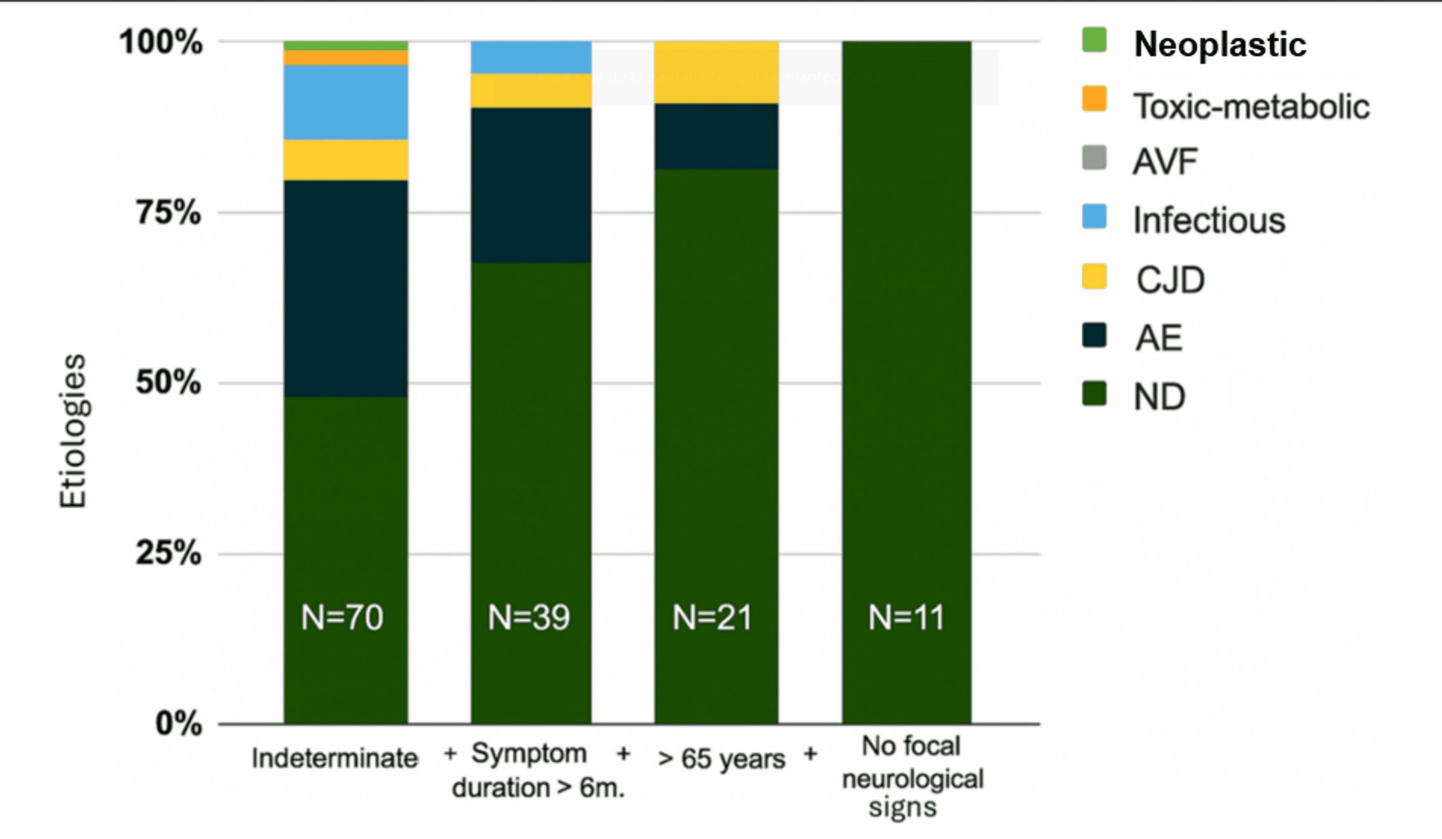
Application of the model in our cohort AE: autoimmune encephalitis; ND: neurodegenerative; CJD: Creutzfeldt-Jakob disease; AVF: arteriovenous fistula Image credits: Francisco Gortari

Finally, the predictive factors were applied to our cohort of 70 patients aged over 65 years, with a symptom duration of more than six months and absence of neurological signs, obtaining a total of 11 patients, all with ND etiology (Figure 4).

## Discussion

Our study has demonstrated that the combined use of clinical and demographic factors and complementary studies allows for the prediction of the etiology of ND diseases in patients with RPD with moderate efficacy. Historically, the study of RPD was focused on prion etiologies, influencing the development of cohorts in which this cause predominated, followed in frequency by ND etiologies [3,7]. However, in these cohorts, autoimmune etiologies were underestimated due to the absence of standardized diagnostic criteria at that time. With the development of diagnostic criteria and the discovery of specific antibodies, autoimmune etiology has acquired a central role in the study of RPD, as early diagnosis and treatment are key determinants of prognosis [14,15]. Current literature suggests that the initial approach should focus on this etiology, combining clinical criteria and imaging findings and, in some cases, using specific predictive models [3,6,14,15].

From a clinical perspective, several "red flags" have been identified that suggest an autoimmune etiology, such as younger age, symptom duration of less than six months, history of recent infection or vaccination, systemic comorbidities, focal neurological signs, and seizures [6,14,15]. Regarding complementary studies, certain imaging patterns can help differentiate the different RPD etiologies: cortical, basal ganglia, and thalamic hyperintensities on DWI for CJD; temporal, insular, and frontal T2 and FLAIR hyperintensities for herpes encephalitis; and mesial temporal and cortical hyperintensities are characteristic of AE [16,17]. However, in many cases, neuroimaging findings are non-specific, making it challenging to distinguish treatable etiologies.

Recently, the STAM3P score has been proposed as a tool to identify treatable etiologies in RPD [6]. This model incorporates factors such as the presence of seizures, suggestive MRI findings of AE, association with tumors, age below 50 years, psychiatric symptoms (mania), abnormal movements, and cerebrospinal fluid (CSF) pleocytosis. A score ≥ 1 has a positive predictive value of 75%, while a score of 3 or higher demonstrates 100% specificity for a treatable etiology [6].

Despite these advances, a standardized diagnostic algorithm is still lacking in determining which patients require an exhaustive antibody screening, which could optimize the diagnostic approach. Our proposal focuses on ND etiology, as it remains one of the most frequent in the studied cohorts [3,7,8]. Furthermore, the development of precise serological biomarkers, such as p-tau 217, 231, and 218, as well as Aβ42 [9,10], combined with the recent approval of disease-modifying therapies, such as lecanemab and aducanumab [11,12,18], opens new opportunities to reconsider the diagnostic strategy in RPD.

Although clinical trials with monoclonal antibodies have demonstrated reductions in disease progression and functional improvements in patients with chronic ND dementia [11], these beneficial effects are most pronounced during the early stages of the disease [18]. In this context, although no specific trials have been conducted in patients with RPD, a therapeutic window similar to that observed in AE may exist, in which treatment initiated during the mild dementia stage, as mentioned previously, could significantly modify the prognosis.

Considering these factors, ND disorders, historically considered a diagnosis of exclusion, could become a central aspect of the diagnostic approach in RPD alongside AE. We propose the development of an algorithm based on a predictive model that allows for the identification of a subgroup of patients in whom the diagnostic approach can be optimized, avoiding unnecessary studies and prioritizing the early identification of a possible ND etiology.

Our study has several limitations. First, the cohort spans from 2013 to the present, which implies that the diagnostic criteria for AE (published in 2016) were not available at the beginning of the study period, and additional antibodies have since been identified [15]. Moreover, revisions to the diagnostic criteria for CJD and the lack of RT-QuIC testing in CSF may have contributed to an underestimation of these etiologies. The relatively small sample size also limits the statistical power of the analysis and restricts the number of variables that can be included in the models [19]. In addition, this was a single-center study, which may limit the generalizability of the findings. Finally, the initial algorithm used for case selection may have inadvertently excluded some false negatives, introducing a potential selection bias.

Our study represents an initial step toward a new approach to patients with RPD, in which clinical and demographic factors may help distinguish ND from other etiologies. Expanding the study to include a larger cohort and refining the criteria used in the predictive model could contribute to the development of a more robust diagnostic tool in the future.

## Conclusions

Despite its limitations, this study may propose a novel and practical approach to identifying probable ND etiologies in patients with RPD. The use of a stepwise diagnostic algorithm (including laboratory testing, neuropsychological screening, and MRI), followed by the application of key predictive factors such as age at onset, symptom duration, absence of neurological signs, and absence of seizures, may facilitate the early recognition of ND causes. This strategy is particularly relevant in the current era of emerging biomarkers and evolving therapeutic options, where ND etiologies, alongside AE, could increasingly be considered as potentially treatable conditions.

This represents an initial approach. Expanding the study with a larger sample size, incorporating additional predictive factors, and leveraging the availability of biomarkers could enhance and refine the criteria used in the predictive model, contributing to the development of a more robust and comprehensive tool.
